# Supplementary material for: Coordination of the N-Terminal Heme in the Non-Classical Peroxidase from Escherichia coli
Source: Molecules. 2023 Jun 7;28(12):4598. doi: 10.3390/molecules28124598 (PMC10301912; doi:10.3390/molecules28124598)
Supplement: Supplementary file 1 [file molecules-28-04598-s001.zip › molecules-2323045-supplementary.pdf]

Supplementary Materials

Coordination of the N-Terminal Heme in the Non-Classical Peroxidase from *Escherichia coli*

Ricardo N. S. Oliveira [1,2,†], Sara R. M. M. de Aguiar [1,2,†], Sofia R. Pauleta [1,2,*]

[1] Microbial Stress Lab, UCIBIO—Applied Molecular Biosciences Unit, Department of Chemistry, NOVA School of Science and Technology, Universidade NOVA de Lisboa, 2829-516 Caparica, Portugal

[2] Associate Laboratory i4HB—Institute for Health and Bioeconomy, NOVA School of Science and Technology, Universidade NOVA de Lisboa, 2829-516 Caparica, Portugal

* Correspondence: sofia.pauleta@fct.unl.pt; Tel.: +351-2129-48385 (ext. 10967); Fax: + 351-212-948-550

† These authors contributed equally to this work.

INDEX

S1. Primers for site-directed mutagenesis p. S2

S2. Primary sequence alignment and structural model p. S2

S3. SDS-PAGE of the isolated proteins p. S3

S4. Visible spectra of YhjA in the presence of cumene hydroperoxide p. S4

S5. Thermostability studied by CD p. S4

S1. Primers for site-directed mutagenesis

The primers used for site-directed mutagenesis are presented in Table S1.

Table S1. Sequence of the primers used for site-directed mutagenesis.

M82A	Forward	TCCTGGCGCGAAACAGTTGGCGGATTA**CGA**CATTAAGCTT
	Reverse	AAGCTTAATGTCGTAATC**CGC**CAACTGTTTCGCGCCAGGA
M125A	Forward	TGAATGGGTGATGCAGTATGAAACTGA**GCC**ACCACCGCGT
	Reverse	ACGCGTTGGTGG**CGC**AGTTTCATACTGCATCACCCATTCA
H134A	Forward	ACGCGTTATACCGCGCTA**GCC**TGGGCGGGT
	Reverse	ACCCGCCCAGGCTAG**CGC**GGTATAACGCGT
C59AC62A	Forward	CTC CGC GAA AAA GGA **GCC** GAC TAT **GCC** CAC ACG CCT TCG GC
	Reverse	GC CGA AGG CGT GTG **GGC** ATA GTC **GGC** TCC TTT TTC GCG GAG

S2. Primary sequence alignment and structural model

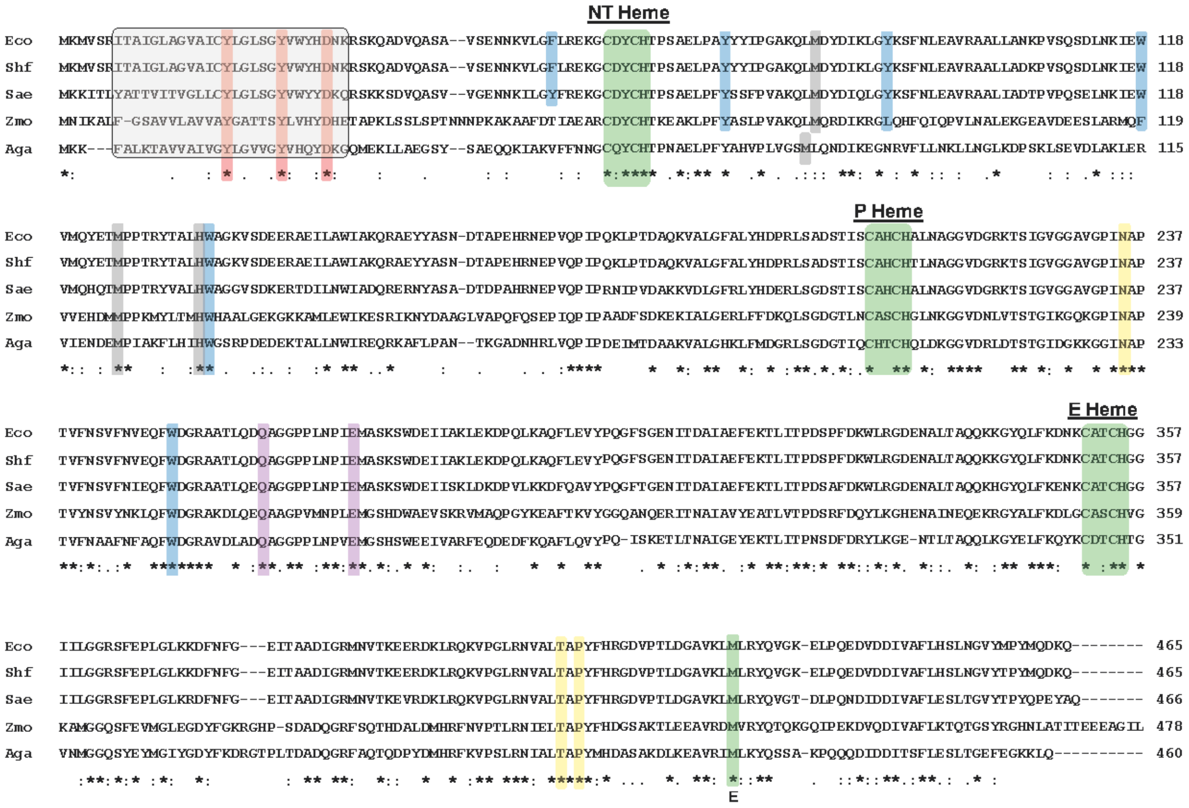

Figure S1. Multiple primary sequence alignment of non-classical bacterial peroxidases. *E. coli* (Eco) (WP_000784836.1), *A. actinomycetemcomitans* (Aga) (WP_005543665.1), *Salmonella enterica* (Sae) (WP_000724476.1), *Z. mobilis* (Zym) (WP_014848281.1), *Shigella flexneri* (Shf) (WP_000784828.1). Homology code: asterisks, colons or stops below the sequence indicate identity, high conservation or conservation of the amino acids, respectively. Consensus sequence for *c*-type heme-binding and distal axial ligand of E heme (E) in green; residues proposed to be involved in semiquinone-protein interactions in red; key residues involved in the catalytic cycle in purple; residues proposed to be involved in electron transfer to and between hemes in blue; calcium binding residues in yellow; residues proposed to be the axial ligand of NT heme in grey; predicted transmembrane helix in a grey box. Sequence alignment performed with ClustalOmega (EMBL-EBI).

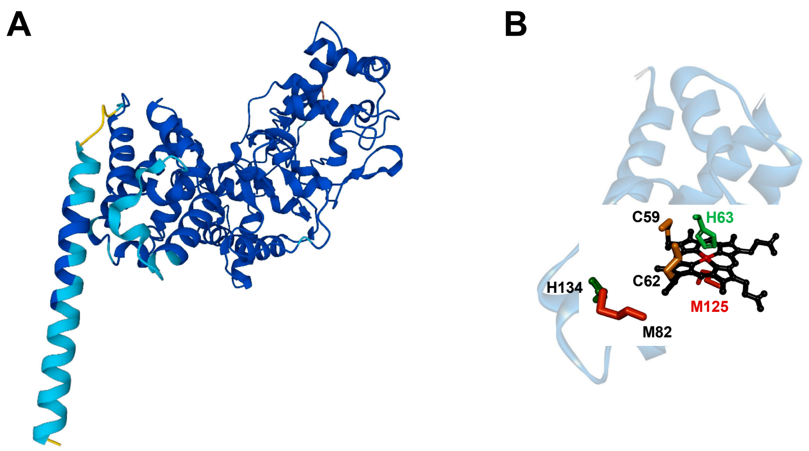

Figure S2. Structural model of YhjA obtained from the AlphaFold2 CoLab. (A) The backbone of the protein is colored by the level of confidence. Per-residue confidence score (pLDDT) between 0 and 100: pLDDT > 90 (very high) in dark blue; 70 < pLDDT < 90 (confident) in light blue; 50 < pLDDT < 70 (low) in yellow. (B) Representation of the backbone of the NT domain with the heme bound to the two cysteine residues through a thioether bound between its thiol group and vinyl heme group, and showing the spatial location of the axial histidine residue and putative axial ligands M82, M125 and H134.

S3. SDS-PAGE of the isolated proteins

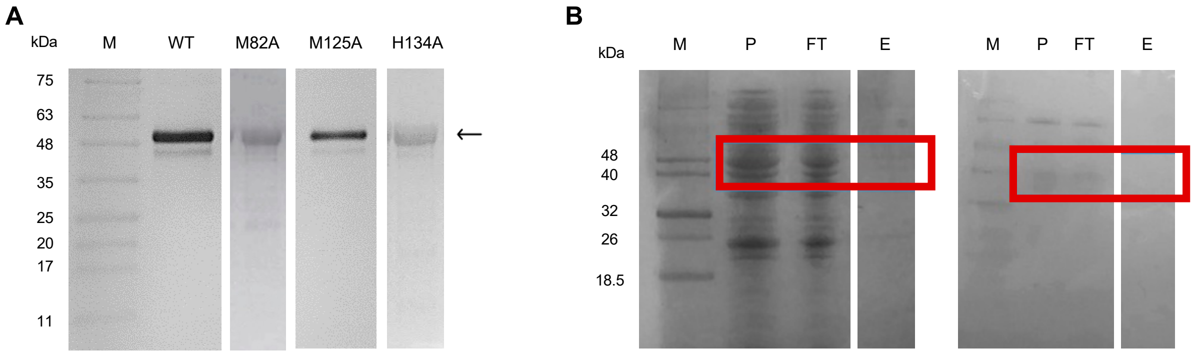

Figure S3. 12.5 % SDS-PAGE of the (A) isolated YhjA WT and variants used in this work, and (B) periplasm of cells expressing the construct of YhjA C59AC62A (P), the flow-through of the column (FT) and its elution with desthiobiotin (E). Gels were stained with Coomassie-blue (A and left gels in B) and for the presence of *c*-type heme (right panel in B).

S4. Visible spectra of YhjA in the presence of cumene hydroperoxide

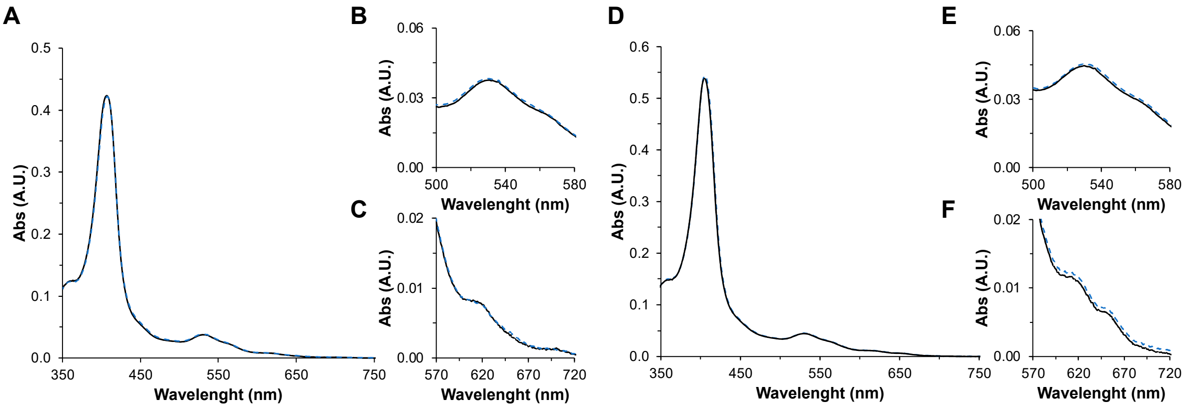

Figure S4. Visible spectra of 2 μM soluble YhjA WT (A-C) and M125A variant (D-F) in the oxidized state, in 20 mM HEPES, pH 7.5, in the presence of 10-fold cumene hydroperoxide. Panel (B) and (E) highlight the Q band region and Panel (C) and (F) the 620 nm band region. Black solid line, enzyme before addition of peroxide; dashed blue line, after incubation with cumene hydroperoxide. Spectra was recorded between 350 nm and 750 nm.

S5. Thermostability studied by CD

The percentage of secondary structure motif was determined for each different spectrum at the different temperatures, for YhjA WT and M125A variant using the BeStSel algorithm.

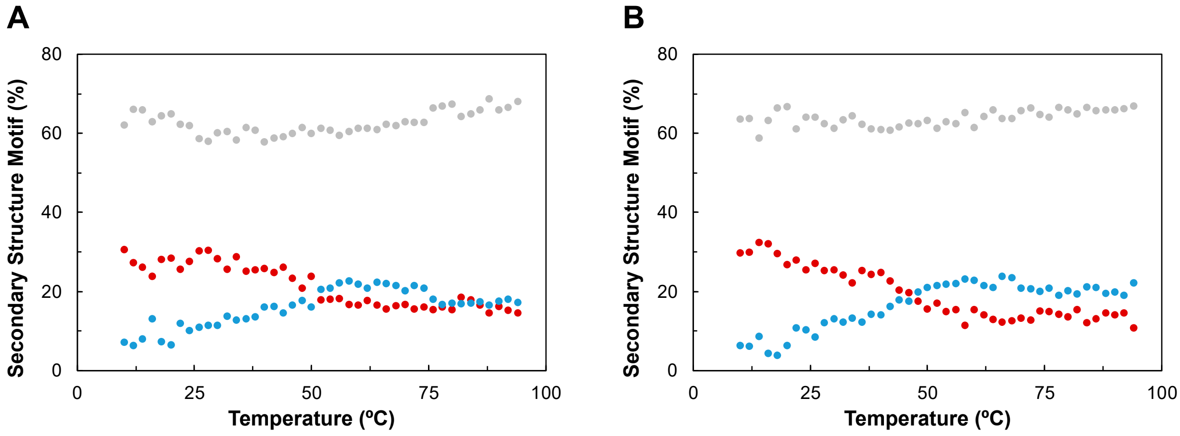

Figure S5. Variation of the percentages of α-helix (red), β-sheets (blue) and other secondary structure motif (grey), with the temperature ramp for the YhjA WT (A) and YhjA M125A (B). The secondary structure content was determined using the BeStSel algorithm.

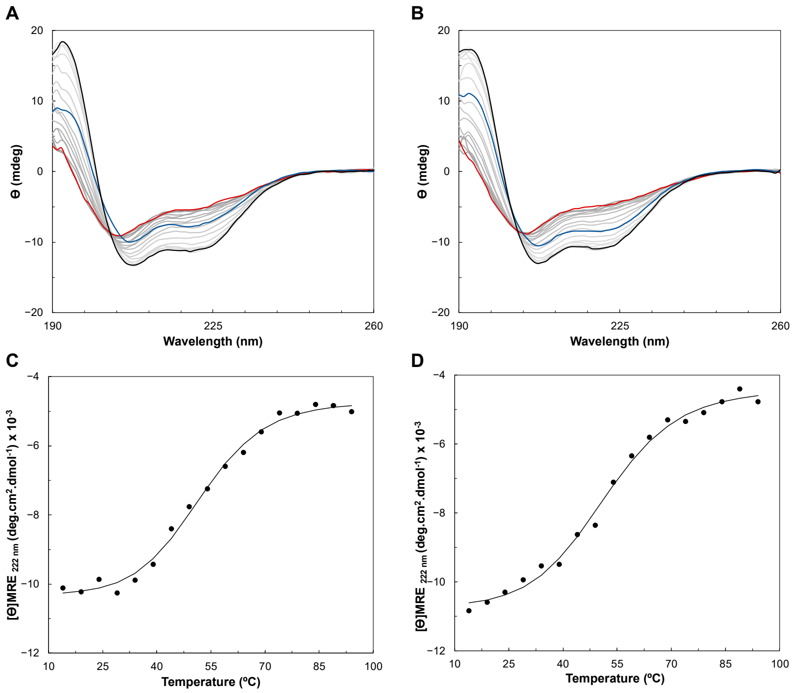

Figure S6. Thermal denaturation of YhjA M82A (Panel A and C) and YhjA H134A (Panel B and D), monitored by circular dichroism in the far-UV region. Panel (A) and (B): Spectra acquired during a temperature ramp between 14 °C (black) and 94 °C (red); spectra acquired at 20 °C after the temperature ramp in blue. Panel (C) and (D): Mean residue ellipticity ($[\theta]_{MRE}$) at 222 nm as a function of temperature for (C) YhjA M82A and (D) YhjA H134A. The data were fitted to a two-state transition according to equation 1 to 4, with a T_m of 51.9 °C and a ΔH of 111 kJ.mol^{-1}, for YhjA M82A and a T_m of 51.4 °C and a ΔH of 98 kJ.mol^{-1}, for YhjA H134A.
